# The role of IL‐22 in the resolution of sterile and nonsterile inflammation

**DOI:** 10.1002/cti2.1017

**Published:** 2018-04-18

**Authors:** Saleh Y Alabbas, Jakob Begun, Timothy H Florin, Iulia Oancea

**Affiliations:** ^1^ Faculty of Medicine School of Clinical Medicine The University of Queensland Brisbane QLD Australia; ^2^ Chronic Disease Biology and Care Group at Mater Research Institute Translational Research Institute The University of Queensland Brisbane QLD Australia

**Keywords:** hepatitis, inflammatory bowel disease, inflammatory diseases, innate lymphoid cells, interleukins, T helper 17 cells

## Abstract

In a broad sense, inflammation can be conveniently characterised by two phases: the first phase, which is a pro‐inflammatory, has evolved to clear infection and/or injured tissue; and the second phase concerns regeneration of normal tissue and restitution of normal physiology. Innate immune cell‐derived pro‐inflammatory cytokines and chemokines activate and recruit nonresident immune cells to the site of infection, thereby amplifying the inflammatory responses to clear infection or injury. This phase is followed by a cytokine milieu that promotes tissue regeneration. There is no absolute temporal distinction between these two phases, and cytokines may have dual pleiotropic effects depending on the timing of release, inflammatory microenvironment or concentrations. IL‐22 is a cytokine with reported pro‐ and anti‐inflammatory roles; in this review, we contend that this protein has primarily a function in restitution of normal tissue and physiology.

## Introduction

Inflammation is a normal physiological response to infection (nonsterile) and/or sterile injury.^1^ The inflammatory response can usefully be divided into two phases: the first phase of a systemic or local inflammatory reaction is to eliminate infections and/or remove damaged tissue; and the second phase is characterised by promotion of tissue repair and restitution of normal physiology once the danger has been cleared. Where there is infection, the adaptive immune system enables memory cells which recognise pathogens, and on a future encounter, will react with a faster and more specific elimination.^1^ On the one hand, the phases of the inflammatory response are not precisely compartmentalised, but on the other hand, there is a temporal sequence of cellular, molecular and physiological events.

The initial phase involves membrane receptors and soluble mediators from damaged cells and cells responding to infection, which signal initially to local resident phagocytic cells and other resident and circulating antigen‐presenting dendritic cells. If sufficiently activated, these cells attract more circulating immune cells that help eliminate damaged or infected cells. Once infection and damage have been cleared, other cellular and soluble mediators can initiate restitution to engender tissue repair.

In primary injury or infection, the inflammatory response to an acute insult is initiated by the activation of innate immune cells. These include resident macrophages (Kupffer cells), natural killer T cells (NKT), innate lymphoid cells (ILCs) and neutrophils. These cells express pattern recognition receptors (PRRs) that detect microbial components, pathogen‐associated molecular patterns (PAMPs, e.g. lipopolysaccharide) or host‐derived damage‐associated molecular patterns (DAMPs) released from injured cells (e.g. mitochondrial DNA).^2^, ^3^, ^4^, ^5^, ^6^ Following this detection, these cells activate a network of signalling pathways and cellular responses, including secretion of multiple cytokines, notably tumor necrosis factor (TNF‐α), interleukin‐1 beta (IL‐1β), interferon‐gamma (IFN‐*y*), IL‐17 and IL‐22 that further recruit, co‐ordinate and activate pro‐inflammatory and regulatory anti‐inflammatory cells.^2^, ^3^, ^4^, ^5^, ^6^

A major question in this field is what determines the evolution of inflammation towards the healing or restitution phase. The initial inflammatory response is associated with pro‐inflammatory cytokines (IL‐1β, IFN‐*y*, IL‐6, TNF‐*α*, IL‐17 and IL‐23), which activate and recruit immunoinflammatory cells to an injury. The latter healing phase is more associated with cytokines that promote healing (IL‐10, IL‐4, IL‐22) although the distinction between pro‐inflammatory and anti‐inflammatory effects is far from clear‐cut with many cytokines appearing to have dual roles (such as IL‐17 and IL‐22). In this review, we will argue that the dual roles may be more apparent than real and will depend on experimental and physiological context, in particular timing and the nature of the injurious insult.

This review focuses on IL‐22. The review will summarise current understanding of the cellular sources of this cytokine, the target cells and the effects of IL‐22 on inflammation in general with a focus on liver injury, and from a review of the literature, argue that the primary role of IL‐22 is in the second restitution phase of inflammation to restore normal tissue.

## Interleukin‐22 (IL‐22)

IL‐22 was initially classified as a cytokine within the IL‐10 family, but more recently, it has been placed within the smaller IL‐20 subfamily, along with IL‐19, IL‐20, IL‐22, IL‐24 and IL‐26.^7^, ^8^ The human *IL‐22* gene encodes a protein of 179 amino acid in length, which is secreted in a form of 146 amino acid polypeptide once a signal peptide of 33 amino acid is cleaved off.^9^, ^10^ IL‐22 has been investigated both in humans and mice. There is an approximately 80% sequence similarity between them.^11^, ^12^ The structure of IL‐22 consists of six alpha‐helices (referred to as helices A to F).^9^, ^13^

IL‐22 is currently being tested experimentally in murine animals as a therapeutic agent via administration with a number of delivery methods including adenovirus expressing IL‐22 and recombinant IL‐22 (rIL‐22). Our studies on wild‐type (WT) mice indicate that exogenous IL‐22 delivered via *intraperitoneal* injections of rIL‐22 has ~30 min half‐life. Exogenous IL‐22 may act locally or systemically, depending on the route of administration and the location of target tissue. In some murine models with metabolic disorders, treatments with a long half‐life IL‐22Fc protein (3 days) have been utilised.^14^, ^15^

## Sources of IL‐22

It has been suggested that sources of IL‐22 or secreting cells in human disease may differ depending on the nature and severity of the disease.^8^ IL‐22 is initially secreted by innate lymphoid cells, part of innate immune response, during early onset of inflammation in response to a primary acute insult where disease has just commenced and at least where there is a first exposure to stimuli.^7^, ^8^, ^16^ The classic source of IL‐22, however, is T‐lymphocyte subsets as part of the adaptive immune response.

### Innate lymphoid cells (ILCs)

Innate lymphoid cells (ILCs) are a recently recognised group of developmentally related innate immune cells.^17^ They share morphological features with T lymphocytes, but do not express recombinant antigen receptors and do not undergo clonal selection and expansion.^18^, ^19^ In addition to regulatory roles in the adaptive immune system mediated through crosstalk between ILCs and classical lymphocytes,^20^, ^21^, ^22^ ILCs express PRRs and respond to a number of other cytokines and chemokines related to inflammatory and infectious stimuli.^18^

ILCs contribute to co‐ordination of inflammatory responses through interactions with both haematopoietic cells, such as macrophages,^23^, ^24^, ^25^ and nonhaematopoietic stromal, endothelial and epithelial cells via secretion of signature inflammatory cytokines such as amphiregulin, IL‐4, IL‐13, IL‐17 and notably IL‐22.^26^, ^27^ The ILCs comprise three main groups: ILC1, ILC2 and ILC3, based on their transcription factor expression pattern and production of cytokines.^28^, ^29^ These groups mirror T‐lymphocyte subtypes T helper 1 (Th1), Th2 and Th17/Th22 that produce signature cytokines TNF‐*α* and IFN‐γ; IL‐4, IL‐5, IL‐9, IL‐10 and IL‐13; and IL‐17 and/or IL‐22, respectively.^30^

IL‐22 is mainly secreted by subsets of ILC3 consisting of three subtypes: lymphoid tissue inducer (LTi) and the other two subtypes that are classified as either positive or negative ILC3 for the natural cytotoxicity triggering receptor (NCR) and divided based on their expression of activating NK cell receptor NKp46 in mice and NKp44 in humans.^31^, ^32^, ^33^, ^34^, ^35^, ^36^, ^37^, ^38^ This group can produce IL‐17RB and/or IL‐22 and depend on the transcription factor RORγt for their development and function.^34^, ^39^, ^40^, ^41^, ^42^ A number of phenotyping studies have determined that NCR^+^ ILC3 expresses IL‐17A^low^ and IL‐22^high^, whereas NCR^−^ ILC3 expresses IL‐17A^high^ and IL‐22^low^ 36, 37, 38.

ILC3s are located in mucosal tissues, predominantly within the gastrointestinal tract, where they are associated with immunity against extracellular pathogens.^31^, ^32^, ^33^, ^34^, ^35^, ^43^ They respond to stimulation with the secretion of one or a combination of classic Th17 and Th22 cytokines,^31^, ^44^ depending on the stimulus. They are likely to be the main lymphocyte where there is primary infection prior to induction of immune memory. They appear to be local sentinels and wardens of tissue homeostasis, being present predominantly at mucosal sites and in nonlymphoid tissues as tissue‐resident cells that expand locally during acute inflammation.^41^, ^45^, ^46^

### Other innate immune

Other innate immune cells such as macrophages, monocytes and mature or immature dendritic cells (DC) do not produce IL‐22,^9^, ^47^, ^48^ and IL‐22 is not produced by nonhaematopoietic tissue cells.^9^, ^48^, ^49^, ^50^

### T helper lymphocytes (Th lymphocytes)

IL‐22 is previously known to be mainly expressed by Th17 cells along with IL‐17 secretion.^51^

Th17 cells and their secreted cytokines appear to drive inflammatory responses in extracellular infection and a number of disorders. Investigations have revealed that these RORγt transcription factor‐expressing cells can be induced by IL‐6 and TGF‐beta (TGF‐β) *in vitro* and IL‐23 *in vivo* to produce IL‐17A, IL‐21 and IL‐22.^6^, ^52^ IL‐22 plays a role in increasing the expression of IL‐23 receptor (IL‐23R) found on Th17 cells and consequently upregulates RORγt and activates signal transducer and activator of transcription 3 (STAT3), a transcription factor that is involved with IL‐22 functions.^53^

An important recent discovery is that of a Th22 subset. Th22 is probably the main source of IL‐22.^54^, ^55^ This newly identified Th22 population may have similarities with the Th17 subset but secretes only IL‐22, hence the name. It has been reported that the Th22 lymphocytes produce almost 50% of IL‐22 produced by all T lymphocytes.^56^ Th22 is found to express CCR4, CCR6 and CCR10 and requires transcription factor aryl hydrocarbon receptor (AHR) for the secretion of IL‐22.^57^, ^58^ The differentiation of Th22 appears to be largely dependent on the signalling of IL‐6 and TNF‐α.^59^, ^60^

Th1 and Th2 lymphocyte subsets do not secrete IL‐22. It has been previously described that Th1 can initiate the activation of STAT4, which can enable the differentiation into Th2 via stimulation of IL‐12.^61^ Th2 cells are characterised by production of anti‐inflammatory cytokines including IL‐4, IL‐5, IL‐10 and IL‐13. They are associated with modulation of allergic responses and clearance of parasitic pathogens. The Th2 effects in hepatic inflammation indicate an anti‐inflammatory function, which promotes tissue repairs, ameliorates inflammation and limits hepatocytes death and apoptosis,^62^ but on the one hand, there is no evidence of IL‐22 secretion by Th2 or Th1 for that matter in liver or elsewhere. On the other hand, there is evidence that another population of T cells, γδ T cells, secrete IL‐22 in mice infected with lymphocytic choriomeningitis virus (LCMV).^60^ Viral infections including LCMV induce the secretion of IL‐22 in spleen, thymus and liver.^60^

## IL‐22 receptor (IL‐22R)

The IL‐22R is a type 2 cytokine receptor consisting of two subunits, IL‐22R1 and IL‐10R2.^63^, ^64^ IL‐22 has been shown to initially bind to IL‐22R1 and then to IL‐10R2.^63^, ^65^ This contrasts with the other members of IL‐10 family including IL‐19, IL‐20 and IL‐24 each of which relies on IL‐10R2 for activation that follows with the activation of STAT, JAK (Janus kinase) and Akt cascades.^9^ IL‐22R1 is known to be the responsible subunit for the binding and activation of STAT3 molecules through its cytoplasmic tail.^10^, ^49^, ^66^ Others have reported that the phosphorylation and recruitment of STAT3 are also dependent on IL‐22 and proper binding of IL‐22R.^63^ For example, lack or dysfunctional IL‐22 was found to result in the disruption and inactivation of STAT3 and downstream pathways in epithelial cells of murine intestine following chemically induced colitis and also *in vivo* in *IL‐22* KO mice.^63^, ^67^, ^68^

The structure of mouse IL‐22R gene and secreted IL‐22 is closely similar and comparable to human component, indicating comparable physiological functions from one model to the other.^64^ As previously mentioned, IL‐22 is produced by ILC3 both in mouse as NKp46 ILC3 and humans as NCR ILC3. Moreover, the expression of IL‐22R is clinically and physiologically important in dictating the role of IL‐22 based on the site the receptor is found on either in human models or animal models. Th17 cells are found on human and mice secreting similar cytokines including IL‐17A, IL‐17F, IL‐21, IL‐22 and IL‐23, as well as similar chemokines including CCL20. Th17 cells express retinoic acid‐related orphan nuclear hormone receptor C (RORC) compared to the mice counterpart (ROR*y*t). However, their expression of IL‐22R and secretion of IL‐22 seem to be similar clinically. For example, Th17 cells and its cytokines have been cast to drive inflammatory responses in patients with inflammatory bowel disease (IBD) and experimental mice models. GWAS (genome‐wide association study) has classified multiple IBD susceptibility genes involved with Th17 such as IL‐12 beta, IL‐23R, STAT3, CCR6 and JAK2.^69^ Overall expression of Th17 cells and the levels of IL‐17A, IL‐17F and IL‐23 were significantly high within the mucosa and lamina propria layer in patients with ulcerative colitis (UC) and Crohn's disease (CD) compared to healthy controls.^70^, ^71^, ^72^ Similarly, the administration of IL‐23 in mice has led to increase in the production of Th17 cells and consequently worsen colitis, whereas anti‐IL‐23 antibodies were able to improve colitis.^73^, ^74^ Similarly, Th22 cells are expressed in mouse and humans but only able to secrete IL‐22 only and do not produce IL‐17. Th22 cells have similar transcription factors to Th17 cells as they express RORC in human tissues and ROR*y*t in mice.^54^

IL‐10R2 cell surface expression is required for the activation of class 2 cytokines, including IL‐10, IL‐22 and IFN‐L1. The antiviral activity of IFN‐L2 and IFN‐L3 is mediated through IFN‐LR1/IL‐10R2 dimer, which stimulates the activation of the JAK/STAT signalling pathway leading to the expression of IFN‐stimulated genes. Polymorphisms of IL‐10R2, one of two dimer receptor ligands of IL‐22, may play a role in modulating HCV disease outcome.^75^

While the biological effect of IL‐22 is believed to be mediated mainly via STAT3 signalling pathway through the binding of IL‐22R1 and IL‐10R2, high concentrations of IL‐22 induce the phosphorylation of other pathways and transcription factors including tyrosine kinases Jak1 and Tyk2. High concentrations of IL‐22 can also activate STAT1, STAT5, MAPKs (mitogen‐activated protein kinase), AKT (protein kinase B, PKB), NF‐κB (nuclear factor‐kappaB) and AP‐1 (activator protein‐1).^76^ This in turn induces other intracellular signalling cascades from MAP kinase pathways including JNK, p38 Kinase and Mek/Erk.^13^, ^66^

### Location of IL‐22R

IL‐22R is mainly expressed by pancreatic islets, intestinal and respiratory epithelial cells and, to a lesser extent, by hepatocytes and keratinocytes.^5^, ^8^ Thus, IL‐22R is well positioned to allow T cells and ILCs, which secrete IL‐22 to regulate proliferation (and influence differentiation more generally) of epithelial cells, endothelial cells and fibroblasts.^63^

IL‐22R is not found on immune cells suggesting that IL‐22 does not regulate the function of these cells.^49^ IL‐22R is rather located at the outer‐body barriers and on parenchymal tissues such as those found on epithelial cells and tissues of respiratory and digestive systems, kidney, skin and liver.^8^ This is consistent with a role of IL‐22 and its receptor in proliferation, resolution and repair of injured tissues that the receptor is found on. Thus, IL‐22R is appropriately expressed in cell subsets in order to control the restitution phase of inflammation.^63^

## Conflicting roles ascribed to IL‐22

It has been reported that IL‐22 and the IL‐22 receptor have dual roles being either protective or pathological in inflammation.^60^, ^63^, ^67^, ^77^, ^78^, ^79^, ^80^, ^81^, ^82^, ^83^, ^84^, ^85^

### Anti‐inflammatory roles

In cerulein‐induced acute pancreatitis, IL‐22 appeared to be an anti‐inflammatory mediator due to its inhibition of inflammatory cell infiltrations mediated by the induction of Reg3 proteins in acinar cells.^86^ For example, on the one hand, injury was prevented via administration of recombinant IL‐22 (1ug/g) 2 hours prior the onset of the acute cerulean‐induced pancreatitis.^87^ (On the other hand, the injury of acute cerulein pancreatitis was not worse in *IL‐22* KO mice;^87^ however, significant inflammatory cell infiltration was observed^86^). In cerulein‐induced chronic pancreatitis, IL‐22 administration with adenovirus ameliorated damage,^86^, ^87^ and IL‐22 deficiency in *IL‐22* KO mice or blocking endogenous IL‐22 with monoclonal anti‐IL‐22 was associated with worse acinar damage.^87^

In a high‐fat diet (HFD) mouse model, IL‐22 played a role in prevention of oxidative and ER stress in pancreatic beta cells via STAT1 and STAT3.^88^ This effect was found to be due to both upregulation of antioxidant genes such as Gpx5 (glutathione peroxidase‐5), Prdx5 (peroxiredoxin‐5) and Sod2 (encoding superoxide dismutase‐2) and downregulation of oxidative stress‐inducing genes: Nos2 (nitric oxide synthase‐2 or iNos), Hsp90ab1 (mitochondrial heat‐shock protein) and Fth1 (ferritin heavy chain‐1). A similar mechanism was also found on intestinal epithelial cells, where a suppression of stress was associated with increased Muc 2 production and restoration of epithelial integrity.^89^

Interestingly in the viral LCMV‐infected mouse model, *IL‐22* KO mice showed hypertrophy of immune organs – thymus and spleen – compared with atrophy following excessive induction of IL‐22 using an adenovirus‐mediated delivery system.^60^

GWAS analysis has identified polymorphisms in IL‐23R in IBD.^90^ A more recent study reported an intestinal protective role of IL‐23R signalling pathway via IL‐22. Mice deficient in IL‐23R in intestinal epithelial cells had impaired mucosal IL‐22 induction in response to IL‐23. Administration of αThy‐1 enhanced the colitis, which was alleviated by IL‐22 treatment, whereas Reg3β administration improved the DSS colitis in these mice by increasing the IL‐22 mucosal levels through recruitment of IL‐22‐producing neutrophils.^91^ Interestingly, a mutation in *IL‐23R* is associated with a reduced likelihood of Crohn's disease. While serum IL‐22 directly correlates with disease activity of Crohn's disease, the lowest serum level was observed in carriers of the minor allele of the p.Arg381Gln mutation, which represents the only coding IL23R SNP and the main CD‐protecting IL23R variant.^92^ These findings taken together are consistent with an anti‐inflammatory role for IL‐22.

### Pro‐inflammatory roles

IL‐22 has been associated with the pathogenesis of a number of autoimmune and inflammatory diseases. A published study reported a correlation between the disease activity of patients with rheumatoid arthritis (RA) and the levels of IL‐22 measured in serum, possibly suggesting a pathogenic function.^84^, ^93^ A pathological function is supported by a significantly less severe form of arthritis in *IL‐22* KO mice with collagen‐induced arthritis.^94^

Similarly, IL‐22 is highly expressed in skin lesions of patients with psoriasis, and IL‐22 levels found in serum were correlated with disease activity.^95^, ^96^ Furthermore, experimental psoriasis was significantly ameliorated in *IL‐22* KO mice or following neutralisation of IL‐22 via anti‐IL‐22 in WT mice.^97^

There are other examples of IL‐22 resulting in adverse outcomes. For example, treatment with recombinant IL‐22 (rIL‐22) exacerbated airway inflammatory responses in mice with acute exposure to cigarette smoke;^98^ at 24 hours following rIL‐22 treatment, there was significantly more pathology in lungs of treated animals.^98^ This pathology was associated with induction of significant pro‐inflammatory cytokines and damaged epithelium of the lung. It is important to note that in this model, the rIL‐22 was administered half an hour before smoke exposure at a dose of 100 mg kg^−1^ per mouse.^98^

Another study has suggested a pro‐inflammatory function of IL‐22 in models of bleomycin‐induced airway inflammation,^99^ in both *IL‐22* KO mice and by anti‐IL‐22 monoclonal antibody in wild‐type mice.^99^ The airway inflammation induced by bleomycin was lethal for WT mice.^99^ *IL‐22* KO mice or neutralised IL‐22 WT mice were ameliorated against bleomycin‐induced disease,^99^ but interestingly, *IL‐17α* KO mice were completely protected against bleomycin, while on the other hand, the administration of IL‐22‐blocking agent in *IL‐17α* KO exacerbated airway inflammation following bleomycin induction, suggesting a local protection of IL‐22 in the absence of IL‐17α.^99^ This was supported by their *in vitro* studies claiming a protection of airway epithelial cells from bleomycin following administration of IL‐22, which was then reversed with co‐administration of IL‐17α.^99^

## Reconciling anti‐inflammatory and pro‐inflammatory roles of IL‐22

### Timing

Timing of an IL‐22 intervention appears critical as shown in the acute cerulein–pancreatitis mouse model.^86^ This is not so surprising because IL‐22 promotes proliferation of epithelial cells, which would be undesirable before infected or otherwise damaged cells in narrow pancreatic ducts were cleared by the inflammatory phase of inflammation. If clearance does not occur, then it is likely that the debris will block the narrow pancreatic ductules resulting in more pancreatitis. A similar explanation may explain the pro‐inflammatory effect of IL‐22 on lung of mice exposed acutely to smoke inhalation.^98^ The presence of large amounts of IL‐22 by promoting proliferation could counter clearance of damaged tissue debris in the inflammatory phase of the model, leading to obstructed small bronchioles.

### The interrelationship with Th17

In the bleomycin mouse model, complete absence of IL‐22 was associated with protection,^99^ but this appeared to relate to the absence of IL‐17α. This is consistent with the requirement for IL‐22 in the evolution of Th17 pro‐inflammatory cells, for example by inducing IL‐23R. In the absence of Th17, IL‐22 is protective. Th17 was presumably absent in the *IL‐17α* KO. Similarly, they are also likely reduced once the first phase of inflammation is exhausted.

### The paradoxical effect with proliferative disease pathologies

Psoriasis is a skin disorder associated with hyperproliferation of keratinocytes. It should therefore not be surprising that a cytokine which promotes proliferation of these cells could exacerbate the pathology or that an *IL‐22* KO animal might be protective of psoriasis.

A similar consideration could apply to rheumatoid arthritis. IL‐22 could conceivably exacerbate arthritis as the disease evolves to irreversible joint deformity.

### IL‐22 dose effects

In the mouse model of acute airway inflammation induced by smoke inhalation, the pro‐inflammatory response followed administration of 100 mg kg^−1^ of rIL‐22.^98^ However, this dose is 1000‐fold >0.1mg kg^−1^ used to induce a protective effect in the high‐fat diet murine model.^88^ It also contrasts with a murine model study of IL‐22's role in *Pseudomonas aeruginosa* lung damage,^100^ where neutralisation of IL‐22 resulted in increased neutrophil infiltration, and lung damage.^100^ The protection in this study followed treatment with 0.1 mg kg^−1^ of rIL‐22 via intratracheal administration 18 hours prior to induction of acute pneumonia with the bacterium.^100^ Similarly, a study of rIL‐22 treatment in an airway disease model triggered by inhalation of methacholine^78^ reported that IL‐22 was protective at the more physiological dose of 0.1–10 mg kg^−1^.^78^ Unlike the smoke inhalation study, the finding of rIL‐22 protection was supported by *IL‐22* KO mice, which developed more inflammation following methacholine challenge.^78^

High‐dose rIL‐22 would activate many other pathways^14^, ^15^, ^101^ including pro‐inflammatory nonphysiological pathways.

### Liver

In liver, the protective function of IL‐22 is mediated by activation of the STAT3 signal pathway and induction of several anti‐apoptotic proteins (such as B‐cell lymphoma 2 (Bcl‐2), B‐cell lymphoma‐extra‐large (Bcl‐xL) and myeloid cell leukaemia 1 (Mcl‐1)). (Mitogenic proteins (such as retinoblastoma‐like protein 2, cyclin D1 and cyclin‐dependent kinase 4 (CDK4)) enhance cellular proliferation during serum starvation, but not under normal growth conditions.^76^, ^83^) IL‐22 may act on liver stem/progenitor cells, which are important for regeneration following liver injury being able to generate hepatocytes and biliary epithelial cells.^2^, ^8^ Consistent with these findings, the total progenitor cell numbers in liver were positively correlated with increased levels of IL‐22 expression in hepatocytes in patients with hepatitis B virus (HBV) or hepatitis C virus (HCV).^2^

The anti‐oxidative effect of IL‐22 has been demonstrated in both acute and chronic murine models of liver injury, where there is increased expression of the antioxidants metallothionein 1 (MT1) and metallothionein 2 (MT2).^76^, ^102^ More recently, it has been demonstrated that IL‐22 can promote nuclear translocation of nuclear factor‐related factor (Nrf2) in an *in vitro* model of alcoholic liver fibrosis (ALF) using hepatic stellate cells to show reduced fibrosis. This effect is proposed to be regulated through induction of the antioxidant signalling cascades, resulting in elevated expression of its downstream target protein glutathione (GSH), and suppression of oxidative stress.^103^

### The role of IL‐22 following sterile insults to liver

In a number of studies, following sterile insults, for example with alcohol or paracetamol (APAP), it appears that IL‐22 acts on both hepatic stellate cells (HSCs) and hepatocytes by promoting proliferation to ameliorate acute liver injury.^104^, ^105^, ^106^ These cells highly express local receptors for IL‐22.

APAP is a widely used over‐the‐counter antipyretic and analgesic medication.^107^ It has well‐known hepatotoxicity if an overdose is taken, causing direct liver injury via the accumulation of toxic metabolites, lowering glutathione in the liver and leading to hepatic damage.^108^, ^109^ This damage or necrosis develops within 3 to 48 hours, which makes it a hyperacute injury.^110^, ^111^ APAP‐induced hepatotoxicity is found to be mainly due to events occurring within the hepatocytes.^112^ The APAP injury is mediated by mitochondrial dysfunction, production of toxic metabolites and ER stress. The injury is in turn amplified by the innate immune system.^109^ Hepatic cell death activates Kupffer cells (hepatic macrophages). These cells produce IL‐12, IL‐18 and TNF‐α that may be responsible for the activation of NKT and NK cells.^109^, ^113^ This activation is believed to drive accumulation and recruitment of neutrophils and other immune cells including increased numbers of activated NKT and NK cells in the liver,^113^ well after the toxin has been catabolised and so increasing the severity of liver damage. Pretreatment with a single dose of recombinant IL‐22 in WT mice significantly reduced the levels of serum alanine aminotransferase (ALT) and histopathologic damage compared with nontreated animals following APAP acute toxicity. Others have shown that this protection of IL‐22 is dependent on STAT3.^114^, ^115^, ^116^

Injudicious alcohol causes a significant liver damage via acute or chronic consumption. Alcoholic liver disease or ALD is caused by prolonged and excessive use of alcohol resulting in alcoholic steatohepatitis and fibrosis where there is an intermittent exposure inducing recurrent episodes of injury and healing. This ultimately leads to cirrhosis.^117^ The initial hepatic injury is the result of oxidative and ER stress and mitochondrial damage induced by alcohol and its metabolites,^118^, ^119^, ^120^, ^121^, ^122^, ^123^ leading to the activation of chemokines that stimulate macrophages, IL‐8 and neutrophils.^124^, ^125^ Kupffer cells,^117^ T lymphocytes and infiltrating macrophages are also linked to ALD. Alcohol also alters gut permeability, which results in high levels of lipopolysaccharide (LPS) in both the hepatic portal vein and systemic circulations. Activation of toll‐like receptor 4 (TLR4) by LPS leads also to activation of a number of inflammatory cytokines.^126^, ^127^

IL‐22 has been shown to ameliorate steatosis and hepatic damage in acute ethanol feeding, chronic‐binge ethanol feeding and high‐fat diet‐induced fatty liver disease.^128^ This occurs with IL‐22 pretreatment in murine models of ALD.^129^, ^130^ Liver damage is also worse in IL‐22‐deficient (*IL‐22* KO) mice.^129^, ^130^, ^131^ Conversely, *in vivo* and *in vitro* studies show liver regeneration or hepatocyte proliferation with overexpression of IL‐22.^128^, ^132^ Similarly, Ki *et al*.^76^ have shown a protection following IL‐22 administration in a murine model of chronic‐binge ethanol feeding. The protection in the chronic model was related to the upregulation of STAT3 pathway as the hepato‐protection was abolished following a genetic deletion of STAT3 in hepatocytes.

While IL‐22 appears to be associated with amelioration of liver injury in hepatic oxidative stress, alcohol‐induced liver injury or HFD models,^76^, ^133^, ^134^ HSCs were also associated with liver fibrogenesis and formation of scar tissue in response to chronic liver injury and expressed high levels of IL‐22R1.^82^ Specifically, IL‐22 administration induced HSCs and prevented their apoptosis *in vivo* and *in vitro*.^82^, ^106^ Furthermore, overexpression of IL‐22 via exogenous administration of expressed IL‐22 via adenovirus or via targeted gene (i.e. transgenic mice for IL‐22) resulted in amelioration of liver fibrosis apparently due to senescence of b‐galactosidase‐positive HSCs.^82^, ^106^ In contrast, a single dose of IL‐22 via the expression of adenovirus induced CXCL1 in the liver attracting circulating neutrophils to cause acute inflammation.^77^

IL‐22 is being investigated as a treatment for acute or chronic liver injury, either by use of IL‐22‐blocking agents or induction of IL‐22‐producing cells.^67^, ^79^, ^102^, ^132^, ^135^ Other approaches include developing small molecule agonists of the aryl hydrocarbon receptor to enable the activation of transcription factors that are responsible for the differentiation of ILC3 and to promote Th22 cells each of which can enhance the production of IL‐22 in the liver.^8^, ^136^ Another alternative is via increasing the presence of IL‐22‐producing cells via blocking agents such as chemokine ligand 20 (CCL20) or liver activation‐regulated chemokine (LARC) or via the use of neutralising antibodies that specifically target IL‐22 and inflammatory mediator cytokines such as IL‐22 or TNF‐*α*.^8^ However, these therapies that are at an early stage of investigation remain controversial because they are associated with high infection rates post‐treatment.

This section of the review focused on acute liver injury via APAP and both acute and chronic liver injuries due to ethanol. Other models of sterile livery injury,^114^ where IL‐22 appears anti‐inflammatory/protective, include carbon tetrachloride (CCL4)‐ and FAS ligand (FASL)‐induced hepatic injury,^137^ and T‐cell‐mediated hepatitis induced by concanavalin A, where there was prevention of the hepatitis in IL‐22 pretreated mice compared with mice injected with IL‐22‐neutralising antibodies.^51^, ^129^ In general, the timing of an IL‐22 therapeutic intervention does not appear to be pro‐inflammatory where the injury model involved a sterile insult. An exception was the acute infiltration of neutrophils with a single dose of 5 × 10^10^ particles of recombinant adenovirus that expressed IL‐22.^77^

### The role of IL‐22 in liver following (nonsterile) infection

In contrast to sterile injuries, a number of studies have indicated that IL‐22 can promote hepatic inflammation in mice models following infection.^5^, ^77^ In these models, IL‐22 appears to exacerbate chronic liver inflammation and fibrosis.^5^ Some of the strongest evidence for a pro‐inflammatory role of IL‐22 is found in models of chronic HBV.^5^, ^85^, ^133^ In patients with chronic HBV, hepatic IL‐22 was highly upregulated and its expression was positively correlated with the severity of injury and inflammation.^2^, ^5^, ^102^, ^129^, ^138^, ^139^ Studies in humans found that Th17‐secreting cells drive the upregulation of IL‐22, and their levels were highly abundant in patients with HBV, associated with increased inflammation and fibrosis.^133^, ^138^, ^140^ In animal models with chronic HBV, IL‐22 is associated with the development of liver fibrosis and cirrhosis.

How to reconcile these pro‐inflammatory outcomes with the hypothesis that IL‐22 is involved in restitution after clearance of infection and damaged cells? The blockade of IL‐22 in the chronic HBV mouse model resulted in reduced Th17 recruitment, which was associated with increased progression of liver inflammation.^5^ While it is unclear whether other immune cells were affected by the blockade of IL‐22 in this study, reduced Th17 could have driven increased inflammation due to impaired clearance of infection. Also *IL‐22* KO mice displayed a significant tissue damage not only in liver.^129^ These outcomes are consistent firstly with the hypothesis that healing responses are impaired in the absence of clearance of infection so leading to fibrosis and cirrhosis, and secondly that absent IL‐22 is associated with reduced restitution after injury.^129^

Others have suggested that the pathological effect of IL‐22 in chronic HBV liver injuries may be due to the activation of IL‐22BP, an inhibitor of IL‐22 preventing the binding with IL‐22R1 and IL‐10R1.^133^ This effect could be abrogated by blocking IL‐22BP to promote resolution of inflammation.^133^ It has been postulated that this activation may be the cause of a number of failed attempts to use IL‐22 injections as a treatment for hepatic inflammation. This receptor may also be activated following high dose of IL‐22 or sustained IL‐22 exposure leading to these adverse effects.

IL‐22 may also be protective against liver fibrosis in human patients with chronic HCV.^135^ This study reported an inverse correlation between the severity and progression of disease and IL‐22 concentration found in cultures from peripheral blood mononuclear cells (PBMC) leucocytes of those patients.^135^ It was concluded that on the one hand, IL‐22 levels were correlated inversely with hepatic fibrosis.^135^ On the other hand, others have reported that IL‐22 was positively associated with severity of liver fibrosis in HCV but administration of IL‐22 promoted proliferation and inhibited apoptosis of LX‐2 human hepatic stellate cells, a major driver for liver fibrosis and inflammation.^141^ Taking these studies together, an interpretation of the human literature, which fits our hypothesis that IL‐22 is protective, is that elevated levels of IL‐22 may be compensatory to ameliorate inflammation rather being a cause of inflammation.^142^

IL‐22 appeared to be protective against liver damage in a primary *Plasmodium chabaudi* malarial infection.^79^ The absence of IL‐22 in *IL‐22* KO mice was associated with increased mortality and hepatic injury during the infection. In contrast, the rates of mortality and liver damage in *IL‐17α* KO mice were not affected in this model.^79^

## Summary

**Figure 1:**
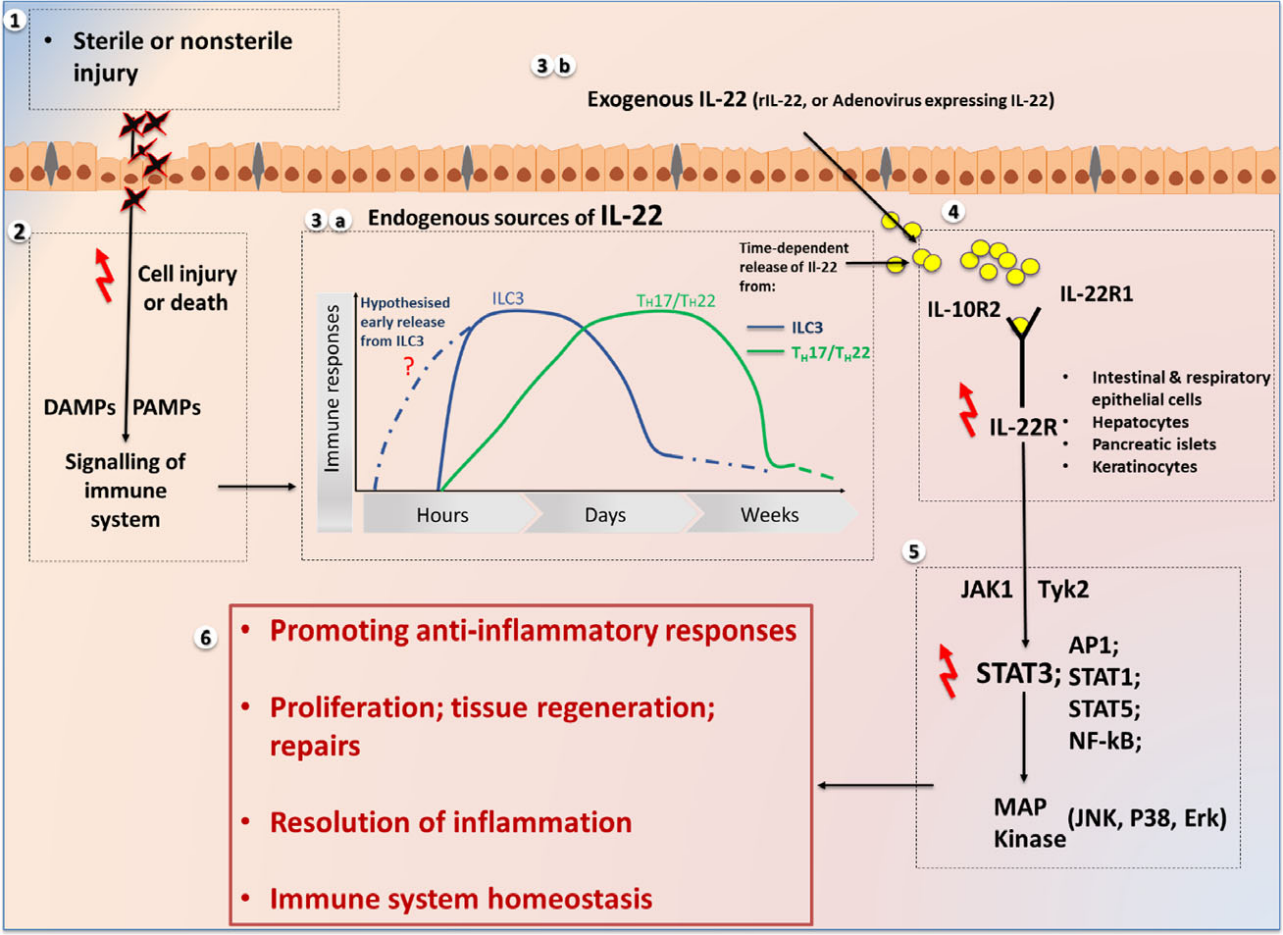
A diagram illustrating a summary of the pathways and roles of interleukin‐22 (IL‐22) following sterile and nonsterile injury. (1) The presence of sterile injury, for example by drugs, or nonsterile injury, for example by infections (2), will lead to cell injury and/or death activating damage‐associated molecular patterns (DAMP) and pathogen‐associated molecular patterns (PAMPs) that signal an immune response. (3)(a) Along with several chemokines and cytokines, endogenous IL‐22 is released from either group 3 innate lymphoid cells (ILC3) and/or Th17/Th22 hours after the induction of injury. It is also proposed that group 3 ILCs (ILC3) might secrete IL‐22 at an earlier stage when compared to TH17/TH22. (3)(B) Alternatively, in experimental‐ or clinical‐based studies, exogenous IL‐22 can be injected directly via recombinant IL‐22 (rIL‐22) or adenovirus expressing IL‐22. (4) Both endogenous and exogenous IL‐22 lead to the binding and activation of IL‐22 receptor (IL‐22R) via its two subunits, IL‐22R1 and IL‐10R2. IL‐22R is commonly found on the intestinal and respiratory epithelial cells, hepatocytes, pancreatic islets and keratinocytes. The initial binding of IL‐22 occurs with the IL‐22R1 subunit, unique to IL‐22 and unlike other cytokines from IL‐10 family, followed by the binding to IL‐10R2. (5) IL‐22 binding to IL‐22R mediates biological effects via phosphorylation of signalling pathways notably via signal transducer and activator of transcription‐3 (STAT3) and activating protein‐1 (AP1), STAT1, STAT5 and nuclear factor‐kappaB (NF‐kB). The signalling from these pathways stimulates the activation of several intracellular signalling processes such as mitogen‐activated protein kinase (MAPK) pathways including c‐Jun N‐terminal kinases (JNK), p38 and extracellular signal‐regulated kinases (Erk). (6) IL‐22 promotes anti‐inflammatory responses following injury by stimulating proliferation, regeneration and repair of injured tissue. IL‐22 thus plays an essential role in the resolution of injury and restoration of homeostasis in the immune system.

In this review, we hypothesise that IL‐22 has a primary role in tissue restitution following clearance of infection and damaged cellular debris from any injury. Th22 lymphocytes typically appear later in inflammation. We contend that pro‐inflammatory effects in humans and in experimental models relate in part to underlying disease pathology (e.g. psoriasis), timing of an exogenous administration of IL‐22 (e.g. where infection is still active) and dose (pharmacological doses of IL‐22 conceivably activate not only STAT3 but also some classical inflammatory pathways) (Figure 1).

Potentially different from Th22, Th17 lymphocytes also infiltrate an injured tissue in the first inflammatory phase. The integrated response of Th17‐ and Th22‐derived cytokines may hold the fate of the inflammatory response. For example, IL‐22 by increasing the expression of IL‐23R on Th17 cells consequently upregulates RORγt and activates STAT3 during the first pro‐inflammatory phase of inflammation.^53^

The roles of Th‐derived IL‐22 versus ILC3‐derived IL‐22 remain largely unelucidated, but it is the same cytokine. Different effects may relate to time of release, with ILC3 playing a more significant role in the early inflammation.
